# Respiratory Complications and Weaning Considerations for Patients with Spinal Cord Injuries: A Narrative Review

**DOI:** 10.3390/jpm13010097

**Published:** 2022-12-31

**Authors:** Kristopher A. Hendershot, Kristine H. O’Phelan

**Affiliations:** 1 Department of Emergency Medicine, Jackson Memorial Hospital, University of Miami, Miami, FL 33136, USA; 2 Department of Neurology, Division of Neurocritical Care, Miller School of Medicine, University of Miami, Miami, FL 33136, USA

**Keywords:** spinal cord injury, respiratory complications, early tracheostomy, predictors of respiratory complications, weaning strategies

## Abstract

Respiratory complications following traumatic spinal cord injury are common and are associated with high morbidity and mortality. The inability to cough and clear secretions coupled with weakened respiratory and abdominal muscles commonly leads to respiratory failure, pulmonary edema, and pneumonia. Higher level and severity of the spinal cord injury, history of underlying lung pathology, history of smoking, and poor baseline health status are potential predictors for patients that will experience respiratory complications. For patients who may require prolonged intubation, early tracheostomy has been shown to lead to improved outcomes. Prediction models to aid clinicians with the decision and timing of tracheostomy have been shown to be successful but require larger validation studies in the future. Mechanical ventilation weaning strategies also require further investigation but should focus on a combination of optimizing ventilator setting, pulmonary toilet techniques, psychosocial well-being, and an aggressive bowel regimen.

## 1. Introduction

Acute traumatic spinal cord injury (SCI) affects approximately 54 people per million in the United States per year, which equates to 18,000 new cases of SCI yearly. Although not the most common type of acute neurotrauma, it has a high rate of long-term disability and an increase in mortality risk of 2–5 times with the highest risk of death occurring in the first year [1,2,3]. Risk factors associated with mortality from SCI include the level of injury, tetraplegia, older age, polytrauma, mechanical ventilation, tracheostomy, septicemia, respiratory complications, and cardiovascular complications [1,2,3].

Respiratory complications, responsible for as many as 42% of deaths, are one of the most common causes of mortality and increased length of stay in patients with SCI [1,4,5,6]. Patients with SCI can develop a range of respiratory complications during the acute, sub-acute, and chronic phases of recovery. Respiratory failure, pneumonia, pleural effusion, pneumothorax, pulmonary embolism, hemothorax, and mucus plugging are common after acute SCI [5]. These complications have been reported to occur more than half of the time in these patients [6]. The prevalence of respiratory failure ranges from 7.8% [7] to 15.4% [8], while the rate of pneumonia has been reported as occurring about one-fifth of the time and is very frequently associated with aspiration [9].

Due to the above respiratory complications, many patients with SCI require advanced airway management at various time points throughout their recovery. Patients with complete cervical SCI, require mechanical ventilation between 81% to 91% of the time [10,11], while patients with incomplete SCI require mechanical ventilation for over 48 h only one-fourth of the time [10]. Many of the patients who are mechanically ventilated will ultimately require tracheostomy as a part of their airway management. There is a wide range, from 12% to 62%, for rate of tracheostomy in the general population of cervical SCI patients [12,13,14,15,16,17]. However, 78% of patients with complete SCI require tracheostomy, while only 15% of patients with AIS D injuries require a tracheostomy [11,12]. The wide range of rates of tracheostomy could indicate a large amount of variability in clinical practice and a lack of clear guidelines for which patients should have a tracheostomy and when it should occur.

In this narrative review, we will discuss common respiratory complications associated with SCI, the underlying pathophysiology, the importance of identifying risk factors for early intervention, and components of weaning from mechanical ventilation that can be integrated into a successful strategy. To ensure that this review included the most relevant and updated literature, we chose to only include publications from the last ten years. We will conclude by discussing two salient cases that will highlight our key teaching points.

## 2. Pathophysiology of Respiratory Complications in SCI

An understanding of the pathophysiology of SCI and how it directly results in respiratory complications is key to our discussion. The level of injury and the strength of accessory muscles play a large role in the development of respiratory complications following a SCI. A cervical SCI may result in a patient who has spontaneous breathing and has a functional diaphragm post injury but produces a forced vital capacity of less than 25% predicted [18]. This leads to hypoventilation due to the inability to fully, or adequately, expand the rib cage during inspiration. Decreased abdominal muscle strength can limit the patient’s ability to produce the forced expiration necessary to cough and clear secretions [19]. Poor lung expansion and secretion clearance leads to the development of pneumonia and mucus plugging and increases the risk of requiring ventilatory support.

Additionally, in SCI, the sympathetic nervous system becomes dormant, while the parasympathetic nervous system becomes upregulated, leading to increased production of airway secretions [7]. These increased secretions, that are then unable to be cleared, can lead to the development of pneumonia and pulmonary edema. Both of which ultimately lead to hypoxic respiratory failure. Respiratory failure may also result from airway obstruction caused by a traumatic or post operative prevertebral hematoma [7]. For patients who undergo surgery, airway compromise could also develop from soft tissue edema or lack of cervical mobility post-operatively [19].

Although a patient may have a stable respiratory status immediately after the injury, and even during the first couple of days of ICU admission, they require close monitoring of their respiratory status as increased secretions, fatiguing respiratory muscles, and increasing atelectasis, can cause worsening hypoxic and hypercarbic respiratory failure days after the initial injury.

## 3. Predicting Respiratory Complications in SCI

Given the prevalence of respiratory complications in patients with SCI, and their often-delayed presentation, it is important for clinicians to identify patients who are high risk. In general, the development of respiratory complications is associated with a history of lung disease, concurrent chest trauma, younger age, sports related SCI, AIS A or B, cervical level of injury, and increased size of injury [5,6]. In older adults with SCI, increased age, higher blood glucose levels, presence of ossification of the posterior longitudinal ligament, presence of a prevertebral hematoma, and an AIS score of A or B were all independent risk factors associated with respiratory complications [7].

The development of pneumonia and respiratory failure has a specific set of risk factors and prediction models to aid clinicians. Within three weeks of injury, patients with complete SCI develop pneumonia about half of the time and are more likely to develop pneumonia if they have underlying medical conditions [11]. Patients with poor Dysphagia Severity Scale scores also have an increased risk of developing pneumonia [9].

Meanwhile, respiratory failure is associated with the level of the SCI, the AIS score, and initial hemoglobin, platelet to lymphocyte ratio, and neutrophil percentage to albumin ratio [8]. A nomogram combining the current gold standard for prediction, level and severity of injury, with biomarkers of inflammatory and nutritional status at the time of injury had an area under the curve of 0.955, which out preformed the gold standard alone [8]. This research group created an online application that clinicians can use at admission to predict patients with SCI who may develop respiratory failure. Although this model has been internally validated with a test group, it needs to be externally validated in a more heterogenous population before it can reliably be used on a large scale.

Patients with SCI are also at higher risk of being unable to be successfully extubated. One recent systematic review found the extubation failure rate was about one-fifth of the time and that it was more likely to occur in patients with complete cervical SCI [20]. Patients with complete injuries, higher injury levels, decreased Glasgow Coma Scores (GCS), increased Injury Severity Scores, and concurrent lung injuries are more likely to require mechanical ventilation for longer than 48 h [10]. Another group found that patients with higher injury severity scores, with a complete or anterior injury, and an injury from C1 to C4 are more likely to require ventilation for 7 or more days [21]. Patients having one of these characteristics had a 67% chance of prolonged ventilation, those with two had an 85% chance of prolonged ventilation, and patients with all three had a 98% chance of prolonged ventilation [21]. The presence of a PAO_2_/FIO_2_ ratio <300 three days after being intubated was also predictive of requiring mechanical ventilation for more than 7 days [14]. Understanding and identifying these risk factors could provide clinicians with an evidence-based rationale for which patients should undergo early elective tracheostomies following a SCI.

Sleep disordered breathing is common and underrecognized in patients with spinal cord injury. This has been studied predominantly in the subacute and chronic phases after injury [22] but likely impacts patients in the acute phase if it was a pre-existing condition. The reported prevalence varies likely due to variable diagnostic methods and criteria. However, many patients exhibit evidence of central sleep apnea as well as obstructive patterns or both. Efforts to successfully identify and treat these patients in the acute setting may improve their overall outcome and quality of life.

## 4. Treatment Considerations for Respiratory Complications of SCI

Most of the respiratory complications associated with SCI can be treated with the accepted standard of care without any special considerations with a few exceptions. While using diuretics or bilevel positive airway pressure to treat a patient with SCI and pulmonary edema or pleural effusions, the clinician must ensure that the patient’s mean arterial pressure remains above the targeted goals. Lastly, due to the increased amount of secretions, and decreased ability to clear them, the management of mucus plugging and atelectasis in patients with SCI is paramount.

In patients who are intubated, direct suctioning of the airway with directional suction catheters that can specifically suction the left lung and frequent bronchoscopy can improve the clearance of secretions. Although not specifically studied in SCI, bronchoscopy has been shown to be effective in resolving lung atelectasis in mechanically ventilated patients and is effective in achieving successful extubation [23]. The use of bronchoscopy to suction mechanically ventilated patients with acute exacerbation of chronic obstructive pulmonary disease has been shown to lead to decreased time on ventilation, decreased hospital length of stay, lower reintubation rates, lower rates of ventilator-associated pneumonia, and higher weaning success rates [24]. The use of mucolytic agents could also play an important role in the management of patients with SCI. However, in mechanically ventilated patients, the routine, combined use of nebulized acetylcysteine with salbutamol did not lead to a decrease in the amount of secretions in the endotracheal tube [25]. More research is needed regarding the use of mucolytic agents and bronchoscopy specifically in patients with SCI.

Many patients with SCI ultimately require definitive airway management for respiratory failure or other respiratory complications that are not easily reversible. Indications for intubation of patients with SCI include acidosis due to an increase in PaCO_2_ or a vital capacity <15 mL/kg [19]. In patients with high, complete cervical SCI, it would be appropriate to consider an elective intubation early in the clinical course [19]. There remains some controversy regarding the appropriate tidal volumes used for patients who are mechanically ventilated. Some suggest that the use of higher tidal volumes may be beneficial in certain patients with isolated SCI and compromised accessory and abdominal muscles [19]. However, a recent study comparing tidal volume settings less than and greater than 15 mL/kg in patients with tracheostomies showed that higher tidal volumes are associated with increased risk of pneumonia and other pulmonary adverse events [26]. This study’s results may not be applicable in most clinical settings as modern protocols for mechanical ventilation generally keep tidal volumes less than 10 mL/kg.

Tracheostomies are common among patients with SCI. In a recent systematic review, patients with the following characteristics were found to be more likely to require a tracheostomy: male, AIS A and B scores, higher level of cord injury, higher injury severity scores, GCS ≤ 8, thoracic injury, and respiratory complications [27]. Patients with lower AIS motor scores, a history of smoking, and patients with a Cervical Spine Injury Severity Score above 7 are also more likely to require tracheostomy [12,13,15]. In fact, the presence of the following factors was predictive of the need for tracheostomy in 95% of patients: 60 years of age or older, combined facet dislocation, C4 level of injury or higher, AIS A or B [16]. Another group developed a classification and regression tree model in order to predict the need for tracheostomy that was 78% sensitive and 96% specific [17]. The model included age over 50 years of age, an Injury Severity Scale score above 16, injury level above C5, and AIS A [17]. This model provides clinicians with a clinical decision tree that can confidently be applied at the individual patient level, although more external validation is required.

Imaging findings may also play a role in predicting who will ultimately require a tracheostomy. The presence of a complete SCI, a level of injury of C5 and above, a maximum canal compromise above 50%, a lesion length greater than 20 mm, and osteophyte formation are MRI findings that are potentially predictive of the need for tracheostomy [28].

In general, tracheostomy leads to improved pulmonary outcomes in patients with SCI [14]. While many patients do ultimately require tracheostomy the correct timing of the procedure requires additional research. Two large systematic review found that early tracheostomy, defined as 7 to 10 days from the injury, produced mixed results. Early tracheostomy was associated with reducing the days on mechanical ventilation, the ICU length of stay, the hospital length of stay, and the risk of ventilator-associated pneumonia and tracheostomy-related complications [29,30]. However, early tracheostomy was not associated with decreased short-term mortality or differences in the rate of developing pneumonia [29,30]. Despite not showing an improvement in mortality, early tracheostomy in selected patients is becoming the standard of care.

## 5. Ventilator Weaning Strategies

The majority of patients with high cervical SCI can be weaned from mechanical ventilation given sufficient time and particularly if they have evidence of diaphragmatic movement. Between 62.6% and 97% of patients who were previously ventilator dependent are eventually able to be weaned at various stages of their recovery [11,13,31,32,33]. One group reported an average of 37 days for weaning [32], although more data about this topic are needed. Complete SCI at C1–C4 is common among patients who ultimately are unable to be successfully decannulated [13].

There are few studies, none that are randomized, regarding specific weaning strategies in patients with acute SCI. One group used a 20 mL/kg tidal volume compared to the standard 10 mL/kg tidal volume while attempting to wean patients off the ventilator over 14 days and found no difference in the days to wean or the number of pulmonary complications [34]. Another group used a combination of theophylline, high volume ventilation, positive pressure treatments, and mechanical insufflation-exsufflation to produce a weaning success rate of 83% [35].

Anecdotally, we have had success with protocols that introduce spontaneous breathing trials for short periods without causing excessive fatigue. The length of trials is slowly increased until the patient is able to tolerate spontaneous breathing modes continuously and can be assessed for extubation or for short periods off the ventilator, if a tracheostomy has been placed. Additionally, we have used testosterone and oxandralone and optimization of nutritional status to increase muscle mass and build strength in order to aid in ventilator weaning. There is some evidence to suggest that building accessory muscle strength can lead to improved outcomes. A meta-analysis of respiratory muscle training showed improved vital capacity, maximal inspiratory pressure, and maximal expiratory pressure in patients with cervical SCI [36]. Further research is needed to identify commonly used weaning strategies in neurocritical intensive care units and then to directly compare them to each other to develop a clinical standard of care.

The inability to clear secretions can contribute significantly to weaning failure. A few important pulmonary toilet techniques that can be integrated into a holistic weaning strategy have been studied in other critically ill patients despite being minimally studied specifically in patients with SCI. Cough augmentation techniques have been studied in critically ill patients and in patients with neuromuscular disorders. Available techniques include manually assisted cough, mechanical insufflation, manual and mechanical breathstacking, mechanical insufflation-exsufflation, and glossopharyngeal breathing [37]. These strategies may lead to an increase in peak cough flow compared to unassisted cough, however none of these techniques has been shown to be superior so individual patient preference may be used to decide the protocol [37]. In patients with neuromuscular disorders, mechanical insufflation-exsufflation may improve peak cough expiratory flow [38]. Additionally, the routine use of mechanical insufflation-exsufflation can lead to improved vital capacity in patients with SCI or neuromuscular disorders [39,40]. Air stacking exercises have also been shown to increase forced vital capacity and peak cough flow in patients with cervical SCI [41]. Combining air stacking, abdominal compression, and spontaneous maximal expiratory effort can lead to near normal peak cough flow [42]. These cough augmentation techniques might help with the clearance of secretions and increase the likelihood of successful weaning from mechanical ventilation.

Although only a few specific weaning strategies have been studied, there is some recent literature pertaining to identification of patients who can be weaned from mechanical ventilation or decannulated. Patients who can be weaned tend to be able to shrug their shoulder within 3 weeks of injury, have higher vital capacities, have higher forced vital capacities, higher maximal inspiratory pressures, and lower spinal level of injury [13,31,43]. Patients who could not be weaned, or had more difficulty being weaned, were over the age of 56, had injuries at C4 or higher, had a vital capacity less than 1500, had a BMI above 25, and had chronic obstructive pulmonary disease [32]. Additionally, patients who are unable to be weaned have a higher likelihood of developing pneumonia and other respiratory complications [31].

Patients who are unable to be weaned from mechanical ventilation but have an intact phrenic nerve are potentially candidates for a diaphragm pacing system. Use of such devices has shown some promise for these patients. One small study showed that 38.1% of patients that were able to be completely dependent on the pacing system were completely weaned from mechanical ventilation [44]. Early implantation of the diaphragm pacing device can lead to patients being able to use the device constantly and no longer require mechanical ventilation [45]. Similarly, transcutaneous electrical diaphragmatic stimulation showed the ability in a small case series to decrease the time on mechanical ventilation and length of stay in the ICU when compared to a hospital’s standard weaning protocol [46].

## 6. Cases Demonstrating Key Aspects of Respiratory Complications and Weaning in SCI

### 6.1. Case 1

**Figure 1:**
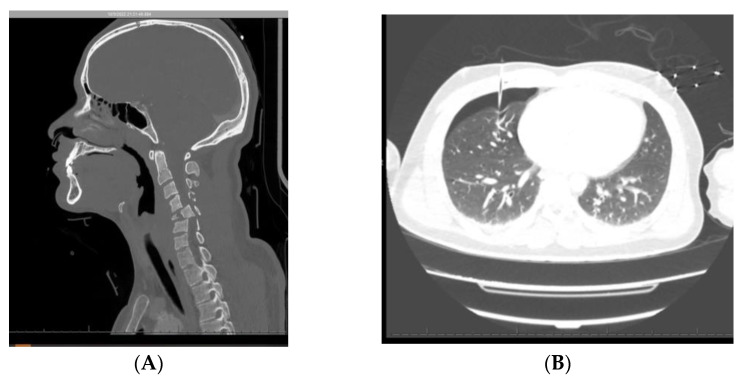

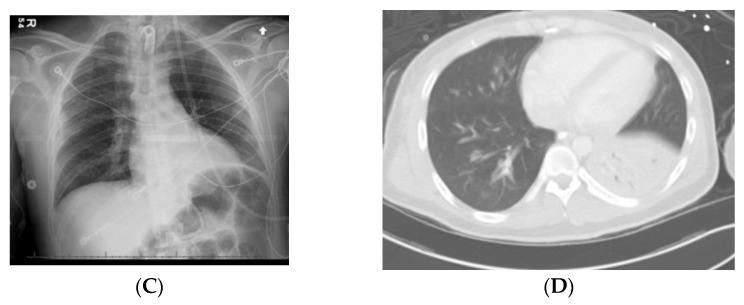
(**A**–**D**). (**A**) CT cervical spine showing C5 burst fracture and area of spinal cord contusion. (**B**) CT chest showing right pneumothorax with decompression needle in place. (**C**) Chest X-ray showing dilated loops of bowel from opioid-induced ileus raising the left side of the diaphragm. (**D**) CT chest showing left lower lobe consolidation and collapse.

A 28-year-old male with no past medical history who was the unrestrained driver in a rollover MVC presented to the trauma center with quadriplegia. The patient was found to have a burst fracture of C5 with severe spinal cord compression resulting in a C4 AIS A injury (Figure 1A). He also had a right pneumothorax that was needle decompressed in the field and later replaced with a chest tube in the trauma bay (Figure 1B). The patient was not intubated until he was in the OR the next day. The patient was immediately started on heated, humidified air through the ventilator and scheduled ipratropium/albuterol, bisacodyl, docusate, and senna. On day 2, the patient developed pneumonia, most likely due to aspiration, and was started on antibiotics. Patient also received his first bronchoscopy on that day, as he had multiple desaturation events, and was found to have a large mucus plug, which was successfully removed. On day 3, the pneumothorax had resolved, and the chest tube was removed. An early, elective tracheostomy was completed on day 5. On day 6, the patient was started on alprazolam, escitalopram, and seen by neuropsychiatry due to concerns about anxiety and depression. Despite the bowel regimen, during the first week of his hospital course the patient developed constipation and an ileus likely from a continuous fentanyl drip for pain and tachypnea (Figure 1C). At that point methylnaltrexone was added to his bowel regimen, which quickly and effectively resolved his ileus. The treatment team attempted to begin weaning the patient off of mechanical ventilation on day 9 with trials of pressure support ventilation. However, the patient poorly tolerated these spontaneous breathing trials due to consistent desaturations and tachypnea. The patient underwent additional bronchoscopies on days 11 and 17 for suctioning due to episodic desaturations and CT findings demonstrating atelectasis, aspiration, mucus plugging, and a persistent pneumonia (Figure 1D). During this time of poor pulmonary function, the team continued to try to decrease the amount of positive end-expiratory pressure required and intermittently trialed pressure support ventilation. The patient had progressively longer pressure support ventilation trials and ultimately was able to be weaned to assist-control ventilation at night and pressure-support ventilation during the day with progressively smaller amounts of pressure support required by the patient. Even with this multimodal weaning strategy, complete weaning for this patient will likely take months.

Multiple factors likely led to difficulty weaning this patient from mechanical ventilation. First, he, like many patients with SCI, developed anxiety and depression revolving around the use of the ventilator. Therefore, optimizing a patient’s mental health with the use of antidepressants and anxiolytic agents is of the upmost importance. The acute loss of autonomy can be overwhelming and counseling by psychologists with SCI experience can be extremely helpful to avoid severe depression and anxiety that often interferes with the ability to fully engage in therapies and advance toward recovery. Specifically undergoing tracheostomy and mechanical ventilation are associated with the development of depression after suffering a SCI [47]. Second, his level of injury has been shown to be associated with difficulty weaning off of ventilator support. Lastly, he also had a concomitant traumatic pulmonary pathology in the pneumothorax and developed a persistent pneumonia in addition to his respiratory failure.

### 6.2. Case 2

**Figure 2:**
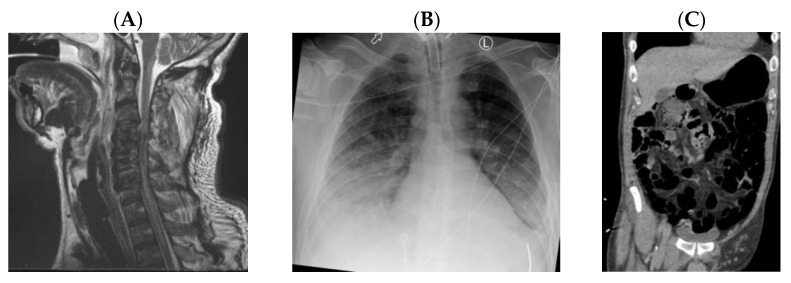
(**A**–**C**). (**A**) MRI cervical spine demonstrating a spinal cord contusion from C4 to C6. (**B**) Chest X-ray showing bilateral basilar consolidations suggestive of pneumonia and bilateral pleural effusions, which is worse on the right compared to the left. (**C**) CT abdomen and pelvis showing multiple loops of dilated bowel concerning for an ileus.

A previously healthy 52-year-old male presented after a helicopter crash with quadriplegia. An MRI showed that the patient had C4 to C6 spinal cord contusions and was diagnosed with a C4 AIS A SCI (Figure 2A). The patient initially had a GCS of 10, L1–L4 fractures, 3 consecutive rib fractures, pneumothorax, vertebral artery dissection, and an open knee wound. On day 0, the patient was intubated for hypoxic respiratory failure and on day 6 he had an early tracheostomy. The patient was immediately started on heated, humidified air through the ventilator and scheduled ipratropium/albuterol, bisacodyl, docusate, and senna. On day 7, he developed pneumonia and was started on antibiotics. This patient also developed an opioid-induced ileus and was given methylnaltrexone, which was effective in resolving the ileus. On day 9, a bronchoscopy was completed and Metanebs were started to help the patient clear secretions. Starting on day 11, the clinical team started trials of pressure support ventilation. During these weaning trials, he developed episodic desaturations and tachypnea. A chest x-ray on day 18 showed developing bibasilar consolidations and pleural effusions requiring treatment with diuretics (Figure 2B). On day 20, the patient began tolerating pressure support ventilation with more ease and only required assist-control ventilation on an as needed basis. However, he once again had difficulty with weaning and developed abdominal distention. A CT of the abdomen showed a moderate stool burden and multiple distended loops of bowel, which were concerning for a new ileus (Figure 2C). After less than a month in the ICU, the patient was transferred to a SCI rehabilitation facility for further weaning and physical therapy.

The patient’s bowel distention likely contributed to his increased work of breathing by causing increase abdominal pressure, which resulted in increased pressure on the thoracic compartment and increased work of breathing. This case highlights the importance of using an aggressive bowel regimen in order to optimize the patient’s intrathoracic and intraabdominal pressures for successful weaning. In a small case series, the use of a spinal cord stimulator to assist with cough was actually found to improve bowel management in patients with SCI, as the time required for bowel management and the use of mechanical methods for bowel management decreased [48]. This patient was also difficult to wean due to his age and due to the presence of rib fractures, which also contributed to his difficulty breathing and increased his risk of developing pneumonia. The patient’s initially decreased GCS likely also contributed to his need for prolonged ventilation and difficulty weaning off of mechanical ventilation.

## 7. Future Directions

Despite the challenges of conducting quality research studies involving patients with SCI and respiratory complications due to the relatively low prevalence compared to other types of neurotrauma, there have been important developments over the last decade in our ability to reliably predict which patients will develop respiratory complications after suffering a SCI. Although many of the developed prediction models have been internally validated, they require larger external validation studies with more heterogenous populations. While individual elements of weaning from mechanical ventilation strategies have been studied to some extent, more research comparing weaning strategies is needed. Specifically, research should focus on extra-pulmonary elements of weaning, such as optimizing patients mental and bowel health. We hope that the next decade of research results in improvements to the existing prediction models and results in a standardized, highly effective mechanical ventilation weaning strategy.

## 8. Conclusions

It is important to understand and acknowledge the underlying pathophysiology of each patient that presents with a SCI in order to reliably predict potential respiratory complications. There remains significant variability among clinicians regarding which patients receive tracheostomy, when they receive them, and how they are weaned from mechanical ventilation. The use of prognostic tools to predict respiratory failure and the need for early tracheostomy could help clinicians develop standardized, evidence-based airway management plans that start at admission. Likewise, mechanical ventilation weaning strategies should be multimodal and holistic in order to address pulmonary and non-pulmonary aspects of weaning.

## Data Availability

Not applicable.

## References

[1] McGrath R., Hall O., Peterson M., DeVivo M., Heinemann A., Kalpakjian C. (2019). The association between the etiology of a spinal cord injury and time to mortality in the United States: A 44-year investigation. J. Spinal Cord Med..

[2] Higashi T., Eguchi H., Wakayama Y., Sumi M., Saito T.M. (2018). Risk factors associated with mortality after traumatic cervical spinal cord injury. OTA Int..

[3] Sengupta D., Bindra A., Kumar N., Goyal K., Singh P.K., Chaturvedi A., Malhotra R., Mishra A.K. (2021). Respiratory morbidity and mortality of traumatic cervical spinal cord injury at a level I trauma center in India. Spinal Cord Ser. Cases.

[4] Josefson C., Rekand T., Lundgren-Nilsson Å., Sunnerhagen K.S. (2021). Respiratory complications during initial rehabilitation and survival following spinal cord injury in Sweden: A retrospective study. Spinal Cord.

[5] Aarabi B., Harrop J.S., Tator C.H., Alexander M., Dettori J.R., Grossman R.G., Fehlings M.G., Mirvis S.E., Shanmuganathan K., Zacherl K.M. (2012). Predictors of pulmonary complications in blunt traumatic spinal cord injury. J. Neurosurg. Spine.

[6] Sampol J., González-Viejo M., Gómez A., Martí S., Pallero M., Rodríguez E., Launois P., Sampol G., Ferrer J. (2020). Predictors of respiratory complications in patients with C5–T5 spinal cord injuries. Spinal Cord.

[7] Ravensbergen H.J., de Groot S., Post M.W., Slootman H.J., van der Woude L.H., Claydon V.E. (2014). Cardiovascular function after spinal cord injury: Prevalence and progression of dysfunction during inpatient rehabilitation and 5 years following discharge. Neurorehabil. Neural. Repair..

[8] Xie Y., Wang Y., Zhou Y., Liu M., Li S., Bao Y., Jiang W., Tang S., Li F., Xue H. (2022). A Nomogram for Predicting Acute Respiratory Failure After Cervical Traumatic Spinal Cord Injury Based on Admission Clinical Findings. Neurocrit. Care.

[9] Hayashi T., Fujiwara Y., Kawano O., Yamamoto Y., Kubota K., Sakai H., Masuda M., Morishita Y., Kobayakawa K., Yokota K. (2022). Incidence and risk factors of pneumonia following acute traumatic cervical spinal cord injury. J. Spinal Cord Med..

[10] Jones T.S., Burlew C.C., Johnson J.L., Jones E., Kornblith L.Z., Biffl W.L., Stovall R.T., Pieracci F.M., Stahel P.F., Moore E.E. (2015). Predictors of the necessity for early tracheostomy in patients with acute cervical spinal cord injury: A 15-year experience. Am. J. Surg..

[11] Liebscher T., Niedeggen A., Estel B., Seidl R.O. (2015). Airway complications in traumatic lower cervical spinal cord injury: A retrospective study. J. Spinal Cord Med..

[12] Menaker J., Kufera J.A., Glaser J., Stein D.M., Scalea T.M. (2013). Admission ASIA motor score predicting the need for tracheostomy after cervical spinal cord injury. J. Trauma Acute Care Surg..

[13] Nakashima H., Yukawa Y., Imagama S., Ito K., Hida T., Machino M., Kanbara S., Morita D., Hamajima N., Ishiguro N. (2013). Characterizing the need for tracheostomy placement and decannulation after cervical spinal cord injury. Eur. Spine J..

[14] Leelapattana P., Fleming J.C., Gurr K.R., Bailey S.I., Parry N., Bailey C.S. (2012). Predicting the need for tracheostomy in patients with cervical spinal cord injury. J. Trauma Acute Care Surg..

[15] Long P., Sun D., Zhang Z. (2022). Risk Factors for Tracheostomy after Traumatic Cervical Spinal Cord Injury: A 10-Year Study of 456 Patients. Orthop. Surg..

[16] Mu Z., Zhang Z. (2019). Risk factors for tracheostomy after traumatic cervical spinal cord injury. J. Orthop. Surg..

[17] Sun D., Zhao H., Zhang Z. (2022). Classification and regression tree (CART) model to assist clinical prediction for tracheostomy in patients with traumatic cervical spinal cord injury: A 7-year study of 340 patients. Eur. Spine J..

[18] Schilero G.J., Bauman W.A., Radulovic M. (2018). Traumatic Spinal Cord Injury: Pulmonary Physiologic Principles and Management. Clin. Chest Med..

[19] Rogers W.K., Todd M. (2016). Acute spinal cord injury. Best Pract. Res. Clin. Anaesthesiol..

[20] Wilson M., Nickels M., Wadsworth B., Kruger P., Semciw A. (2020). Acute cervical spinal cord injury and extubation failure: A systematic review and meta-analysis. Aust. Crit. Care.

[21] Scantling D., Granche J., Williamson J., Gracely E., Thosani D., McCracken B. (2019). Development of clinical tracheostomy score to identify cervical spinal cord injury patients requiring prolonged ventilator support. J. Trauma Acute Care Surg..

[22] Sankari A., Vaughan S., Bascom A., Martin J.L., Badr M.S. (2019). Sleep-Disordered Breathing and Spinal Cord Injury: A State-of-the-Art Review. Chest.

[23] Verma A., Sim W.Y., Tai D.Y., Goh S.K., Kor A.C., Phua C.K., Ho B., Lim A.Y., Lew S.J., Xu H. (2016). Role of Bronchoscopy in Prompt Discharge From the Intensive Care Unit. J. Bronchol. Interv. Pulmonol..

[24] Qiao Z., Yu J., Yu K., Zhang M. (2018). The benefit of daily sputum suction via bronchoscopy in patients of chronic obstructive pulmonary disease with ventilators: A randomized controlled trial. Medicine.

[25] van der Hoeven S., Ball L., Constantino F., van Meenen D.M., Pelosi P., Beenen L.F., Schultz M.J., Paulus F. (2020). For the NEBULAE-investigators Effect of routine vs on-demand nebulization of acetylcysteine with salbutamol on accumulation of airway secretions in endotracheal tubes: Substudy of a randomized clinical trial. Intensiv. Care Med. Exp..

[26] Korupolu R., Stampas A., Uhlig-Reche H., Ciammaichella E., Mollett P.J., Achilike E.C., Pedroza C. (2021). Comparing outcomes of mechanical ventilation with high vs. moderate tidal volumes in tracheostomized patients with spinal cord injury in acute inpatient rehabilitation setting: A retrospective cohort study. Spinal Cord.

[27] Wang Y., Guo Z., Fan D., Lu H., Xie D., Zhang D., Jiang Y., Li P., Teng H. (2018). A Meta-Analysis of the Influencing Factors for Tracheostomy after Cervical Spinal Cord Injury. BioMed Res. Int..

[28] Jeong T.S., Lee S.G., Kim W.K., Ahn Y., Son S. (2018). Predictive Values of Magnetic Resonance Imaging Features for Tracheostomy in Traumatic Cervical Spinal Cord Injury. J. Korean Neurosurg. Soc..

[29] Foran S.J.B., Taran S., Singh J.M., Kutsogiannis D.J.M., McCredie V.M. (2022). Timing of tracheostomy in acute traumatic spinal cord injury: A systematic review and meta-analysis. J. Trauma Acute Care Surg..

[30] Mubashir T., Arif A.A., Ernest P., Maroufy V., Chaudhry R., Balogh J., Suen C., Reskallah A., Williams G.W. (2021). Early Versus Late Tracheostomy in Patients With Acute Traumatic Spinal Cord Injury: A Systematic Review and Meta-analysis. Anesth. Analg..

[31] Korupolu R., Uhlig-Reche H., Achilike E.C., Reeh C., Pedroza C., Stampas A. (2022). Factors Associated With Ventilator Weaning Success and Failure in People With Spinal Cord Injury in an Acute Inpatient Rehabilitation Setting: A Retrospective Study. Top. Spinal Cord Inj. Rehabil..

[32] Füssenich W., Araujo S.H., Kowald B., Hosman A., Auerswald M., Thietje R. (2018). Discontinuous ventilator weaning of patients with acute SCI. Spinal Cord.

[33] Kornblith L.Z., Kutcher M.E., Callcut R.A., Redick B.J., Hu C.K., Cogbill T.H., Baker C.C., Shapiro M.L., Burlew C.C., Kaups K.L. (2013). Mechanical ventilation weaning and extubation after spinal cord injury: A Western Trauma Association multicenter study. J. Trauma Acute Care Surg..

[34] Fenton J.J., Warner M.L., Lammertse D., Charlifue S., Martinez L., Dannels-McClure A., Kreider S., Pretz C. (2016). A comparison of high vs standard tidal volumes in ventilator weaning for individuals with sub-acute spinal cord injuries: A site-specific randomized clinical trial. Spinal Cord.

[35] Zakrasek E.C., Nielson J., Kosarchuk J.J., Crew J.D., Ferguson A., McKenna S. (2017). Pulmonary outcomes following specialized respiratory management for acute cervical spinal cord injury: A retrospective analysis. Spinal Cord.

[36] Berlowitz D.J., Tamplin J. (2013). Respiratory muscle training for cervical spinal cord injury. Cochrane Database Syst. Rev..

[37] Morrow B., Argent A., Zampoli M., Human A., Corten L., Toussaint M. (2021). Cough augmentation techniques for people with chronic neuromuscular disorders. Cochrane Database Syst. Rev..

[38] Morrow B., Zampoli M., van Aswegen H., Argent A. (2013). Mechanical insufflation-exsufflation for people with neuromuscular disorders. Cochrane Database Syst. Rev..

[39] Stehling F., Bouikidis A., Schara U., Mellies U. (2015). Mechanical insufflation/exsufflation improves vital capacity in neuromuscular disorders. Chronic Respir. Dis..

[40] Bach J., Saporito L., Shah H., Sinquee D. (2014). Decanulation of patients with severe respiratory muscle insufficiency: Efficacy of mechanical insufflation-exsufflation. J. Rehabil. Med..

[41] Jeong J.-H., Yoo W.-G. (2015). Effects of air stacking on pulmonary function and peak cough flow in patients with cervical spinal cord injury. J. Phys. Ther. Sci..

[42] Torres-Castro R., Vilaró J., Vera-Uribe R., Monge G., Avilés P., Suranyi C. (2014). Use of air stacking and abdominal compression for cough assistance in people with complete tetraplegia. Spinal Cord.

[43] Kim T.W., Yang J.H., Huh S.C., Koo B.I., A Yoon J., Lee J.S., Ko H.-Y., Shin Y.B. (2018). Motor and Sensory Function as a Predictor of Respiratory Function Associated With Ventilator Weaning After High Cervical Cord Injury. Ann. Rehabil. Med..

[44] Wijkstra P.J., van der Aa H., Hofker H.S., Curto F., Giacomini M., Stagni G., Agullo M.A.D., Casanoves F.X.C., Benito-Penalva J., Martinez-Barenys C. (2022). Diaphragm Pacing in Patients with Spinal Cord Injury: A European Experience. Respiration.

[45] Onders R.P., Elmo M., Kaplan C., Schilz R., Katirji B., Tinkoff G. (2018). Long-term experience with diaphragm pacing for traumatic spinal cord injury: Early implantation should be considered. Surgery.

[46] Duarte G.L., Bethiol A.L., Ratti L.D.S.R., Franco G., Moreno R., Tonella R.M., Falcão A.L.E. (2021). Transcutaneous electrical diaphragmatic stimulation reduces the duration of invasive mechanical ventilation in patients with cervical spinal cord injury: Retrospective case series. Spinal Cord Ser. Cases.

[47] Matsuda Y., Kubo T., Fujino Y., Matsuda S., Wada F., Sugita A. (2016). Relationship Between Depressive State and Treatment Characteristics of Acute Cervical Spinal Cord Injury in Japan. J. Epidemiol..

[48] DiMarco A.F., Geertman R.T., Tabbaa K., Nemunaitis G.A., Kowalski K.E. (2021). Effects of Lower Thoracic Spinal Cord Stimulation on Bowel Management in Individuals With Spinal Cord Injury. Arch. Phys. Med. Rehabil..

